# Diabetic Retinopathy in Children with Type 1 Diabetes—Occurrence and Screening Using Optical Coherence Tomography

**DOI:** 10.3390/life11060590

**Published:** 2021-06-21

**Authors:** Marta Wysocka-Mincewicz, Joanna Gołębiewska, Andrzej Olechowski, Mieczysław Szalecki

**Affiliations:** 1 Clinic of Endocrinology and Diabetology, Children’s Memorial Health Institute in Warsaw, 04-730 Warszawa, Poland; mszalecki@wp.pl; 2 Department of Ophthalmology, Military Institute of Aviation Medicine, 01-755 Warsaw, Poland; joanna.golebiewska@wp.pl; 3 Faculty of Medicine, Lazarski University, 02-662 Warsaw, Poland; 4 Ophthalmology Department, James Cook University Hospital, Middlesbrough TS4 3BW, UK; olechowski@gmail.com; 5 Collegium Medicum, Jan Kochanowski University, 25-369 Kielce, Poland

**Keywords:** diabetic retinopathy, children, type 1 diabetes, OCT, optical coherence tomography, screening

## Abstract

Purpose: To describe the occurrence of diabetic retinopathy, the principles for pediatric care of patients with diabetes, and the utility of optical coherence tomography. Pediatric patients with type 1 diabetes should be screened for diabetic retinopathy upon the lapse of 5 years following the diagnosis. The patients in the time of puberty, who should be screened promptly after the diabetes diagnosis, and patients with type 2 diabetes are the exceptions. Special attention must be paid not only to retinopathy, but also to other possible concomitant conditions, such as cataract, refractive errors, or neuropathy. New techniques, such as optical coherence tomography angiography (OCTA), may contribute greatly to the early detection of retinopathy, facilitating the decision to modify the treatment. The application of modern insulin pumps with continuous glucose monitoring systems has greatly diminished the incidence rate of early symptoms of diabetic retinopathy in the pediatric population.

## 1. Introduction

Diabetes represents a group of metabolic diseases characterized by hyperglycemia resulting from defects in insulin secretion, insulin action, or both. Amongst children, diabetes is the third most frequent chronic disease, and autoimmune type 1 diabetes (T1D) is the most often. Chronic hyperglycemia causes the failure and impairment of organs such as the eyes, kidneys, nerves, or heart. Diabetic eye disease covers all diabetes complications affecting the eyes, out of which diabetic retinopathy (DR) is the most severe one that carries the risk of blindness, besides cataract and secondary glaucoma that are the most important clinical entities.

The Diabetes Control and Complications Trial (DCCT) and the Epidemiology of Diabetes Interventions and Complications (EDIC) studies have demonstrated that intensive diabetes management provides a significant diminishing risk of developing DR [1,2].

### 1.1. Incidence of Diabetic Retinopathy in Pediatric Population

Nowadays children are within the group of low risk of developing DR; however, the related literature refers to the cases of adolescents with diabetic macular oedema, or even proliferative DR (PDR) [3,4,5,6]. The prevalence of DR in the pediatric population was shown in range between 2.3% and 57.6% [7]. In the study of 4172 patients with diagnosed T1D from 12 years of age, the background DR was 26.7%, initial DR was 10.7%, and proliferative DR was 4.1% [8]. According to the questionnaires surveyed by the T1D Exchange Clinic Registry (*n* = 12,535, mean age of 12.64 years, mean diabetes duration of 5.64 years, mean average HbA1c of 8.6%), the treatment for DR was self-reported by 45 subjects (0.36%), but given the medical record, none of them were actually treated for DR [9]. The treatment for DR is extremely rare amongst children enrolled for the T1D Exchange Clinic Registry. Those findings support the results of Huo et al., who found that screening for DR amongst the youth with type 1 diabetes, solely based on age, and diabetes duration, may be unjustified [10]. The retrospective analysis of 143 patients aged 12 or younger, who attended the diabetic eye screening for the first time within the framework of the Diabetic Eye Screening Programme in Birmingham, Solihull and the Black Country area, reveals that of the children under 12 years of age, 7/73 (9.6%, mean diabetes duration of 7 years) had background DR (BDR). The youngest patient with DR was 8 years old [11]. Among those at the age of 12 years, 5/70 (7.1%, mean diabetes duration of 8 years) had BDR. No patient developed DR before a 6-year diabetes duration in either group. The 20-year analysis of 1604 adolescents with T1D shows that the prevalence of retinopathy has continued to decrease, resulting from improved glycemic control [12]. The authors found that retinopathy was diagnosed in approximately 50% of adolescents with T1D, after a median diabetes duration of 9 years in the early 1990s, as compared with only 12% in recent years. Nowadays, lower-than-expected prevalence and severity of DR has been noticed, moderate non-proliferative diabetic retinopathy has been diagnosed in 10% of cases, and only one person has been treated for proliferative DR (PDR) before the lapse of a 14-year diabetes duration [13]. The extensive study, within the framework of the English screening programs and the Scottish, Welsh and Northern Irish programs, indicates 2125 children with diabetes to have been screened for the first time at the age of 12 or 13 years. Among those diagnosed with diabetes at the age of 2 years, or younger, the incidence rate of retinopathy in one or both eyes equals 20% and 11%, respectively, decreasing to 8% and 2% among those diagnosed between 2 and 12 years of age. Only three children (aged 8, 10 and 11 diagnosed with diabetes) have had images graded in terms of degrees of referable retinopathy, and out of them, two have had not referable diabetic retinopathy at all the subsequent screenings [14]. However, both the US studies [6] and our own [15] have not detected any case of DR.

### 1.2. Risk Factors for Diabetic Retinopathy

Hyperglycemia, diabetes duration, children and youth obesity, puberty, arterial hypertension, hyperlipidemia, and genetic predisposition [16] constitute the most important risk factors of DR in the pediatric population. Puberty is referred to as a one of the main risk factors of developing and progressing retinopathy, whereas the risk of developing DR in the case of patients under 10 years old is minimal and diabetic proliferative retinopathy does not occur at this age [17,18,19]. The duration of diabetes after menarche was associated with a 30% excess risk of DR compared with disease duration before menarche. DCCT revealed hyperglycemia as the strongest cause of late diabetes complications [1], and then it was confirmed in many other studies [20,21,22], but no less important is the duration of these metabolic perturbances. In the pooled analysis from 35 global population-based studies, Yau et al. were shown that for glycated hemoglobin (HbA1c) ≤ 7.0% prevalence of any type of DR was 18.0%, compared to 51.2% in patients with HbA1c > 9.0% [22]. A large study from the US has indicated an increase of 20% (95% CI 6–35%) of the DR risk for every 1-point increase in HbA1c in children with T1D [23]. The prevalence of DR increased dependently on the diabetes duration from 21.1% in patients under 10 years of disease, in comparison to 76.3% in those with ≥20 years duration. A higher mean diastolic blood pressure was revealed as more important than systolic in the risk of DR [21]. Even an elevated albumin excretion rate (AER) is the risk factor of DR observed in studies, so it should be rather considered as a comorbidity, or coexistent complication. However, the appearance of AER should prompt an accurate eye diagnostic.

### 1.3. Classification

Diabetic retinopathy, due to its stages of progression, classified by the Early Treatment of Diabetic Retinopathy Study (ETDRS) [24,25], is presented in Table 1.

Any patient with two or more of the characteristics of severe NPDR is considered to have very severe NPDR.

Proliferative diabetic retinopathy could causing blindness as a result of the following:

- Recurrent vitreous hemorrhages from neovascular vessels;
- Retinal detachment complicating proliferative vitreo-retinopathy;
- Severe glaucoma.

PDR may be classified as high-risk and non-high-risk.

### 1.4. Screening Recommendations

Despite the relatively low risk of developing DR in the pediatric population, the screening guidelines for the post-DCCT era have remained largely unchanged. The current American Diabetes Association guidelines for adolescents with type 1 diabetes suggest an annual, initial examination of dilated eyes at the beginning of puberty, or at age 11, whichever comes first, when a young person has had diabetes for 3 to 5 years [26]. After the initial examination, ADA suggests repeating the dilated and comprehensive eyes examination every 2 years. Less-frequent examinations, every 4 years, may be acceptable on the advice of an eye care professional and based on risk factor assessment, including a history of glycemic control with HbA1c < 8%. The recommendations of the National Institute for Clinical Excellence (NICE) suggest starting screening for DR from 12 years, and annually thereafter [27].

The recommendations of the International Society for Paediatric and Adolescent Diabetes (ISPAD) [28], with respect to the pediatric population, are as follows:

- Screening for DR should start from age 11 with 2 to 5 years of diabetes duration; screening should be performed by an ophthalmologist, optometrist, or a trained experienced observer through dilatated pupils via bio-microscopy examination or fundus photography;
- In the case of children with diabetes duration <10 years, mild non-proliferative retinopathy (only microaneurysms) and a good glycemic control, it is sufficient to have a medical check-up every two years (biomicroscopy, or a color fundus photography);
- Due to the risk of developing retinopathy in children who undergo rapid improvement of treatment after prolonged poor glycemic control, an ophthalmological examination is recommended prior to such treatment and then every three months for one year;
- In the case of retinopathy stages carrying the risk of blindness (severe non-proliferative retinopathy or worse and/or diabetic macular edema), laser photocoagulation and intravitreal injections of anty—vascular endothelial growth factor (anty–VEGF) are recommended, which statistically reduce the risk of vision loss;
- Intensive education and treatment should be implemented to prevent or delay the beginning of vessel problems.

**Figure 1:**
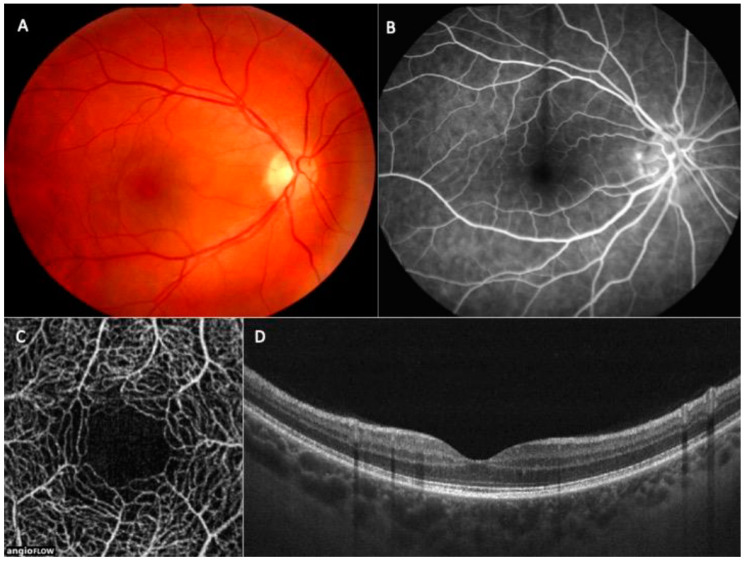
Multimodal imaging of the patient without diabetic retinopathy, (**A**)—color photo of the fundus, (**B**)—fluorescein angiography, (**C**)—optical coherence tomography angiography, (**D**)—OCT B-scan.

The fundamental method for diagnosing DR is a fundus examination with dilated pupils. It is preferable to take pictures using the fundus camera and file them in a patient’s dossier. Fluorescein angiography is a method confirming diagnosis; however, due to its invasive nature, difficulty, and stress for children it is not a routine one in the pediatric population [4,29,30], even in the early stages of DR [31]. Optical coherence tomography (OCT) and optical coherence tomography angiography (OCTA) are rapid, non-invasive methods that may be used in the detection of early diabetic complications in the retina, choroid, or optic nerve; however, the related literature hardly makes any references to children, but instead, adults [32,33,34]. OCT is an invaluable diagnostic tool in the diagnostic of both anterior and posterior segments of the eye. OCTA enables the assessment of the distinct vascular layers of the retina, superficial capillary plexus (SCP) and deep capillary plexus (DCP), comparable to the histologic pictures in Figure 1.

In diabetes, OCTA showed larger foveal avascular zone (FAZ) and early capillary non-perfusion [35]. However, OCTA has some limitations, as it only provides images of the central retina, not its periphery, it requires a few seconds of good fixation, which could be problematic for young children, and because of that the images may be unsatisfactory.

### 1.5. OCT in Paediatric Population

**Figure 2:**
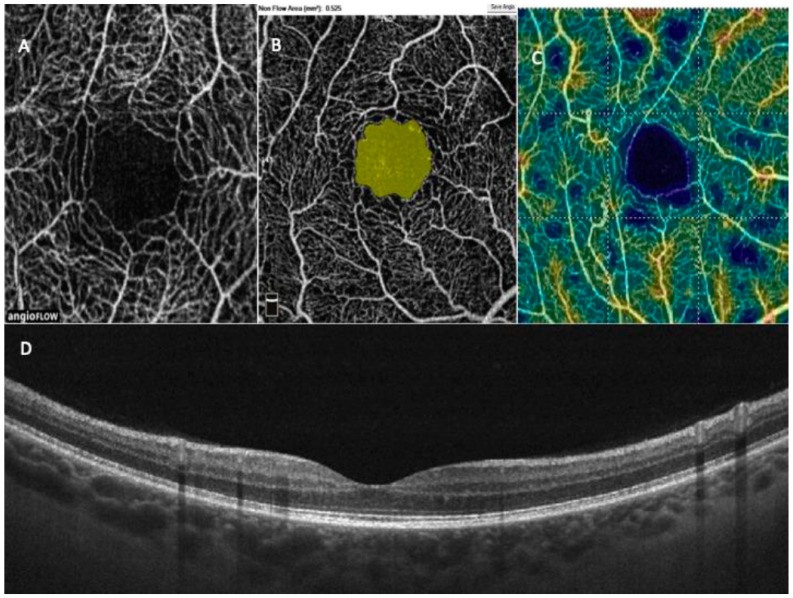
Representative OCT and OCTA images of a child with T1D. (**A**)—superficial retinal plexus with an enlarged FAZ area in the center (scan 3 × 3 mm); (**B**)—automatic measurement of FAZ area (yellow)—the result is visible in the upper left corner (mm^2^); (**C**)—superficial vessel density map—navy blue areas represent non-perfusion zones; (**D**)—OCT B-scan of normal retina, without symptoms of DR.

As we mentioned earlier, retinopathy is extremely rare in the pediatric population, but we need to regularly monitor the very early stages of the disorder to prevent progression and vision loss. Systematic eye examination is especially important in adolescence because, as Wang et al. [23] has shown, DR appears to progress rapidly in this group. The studies in children with T1D have showed an increased central choroidal thickness [36,37] compared to the control group, which increases with the duration of diabetes. Therefore, this parameter may be useful for screening. Vascular density was decreased in children with T1D (*n* = 53) compared to healthy subjects, in both the superficial and deep plexuses except of the fovea [38]; however, these results were not confirmed in another study in the group of 94 children [15]. These results are promising, as OCTA can identify early microvascular changes in the retina that appear before clinically apparent DR, which was undetectable with the standard screening procedures (Figure 2).

Gołebiewska et al. have demonstrated the correlation of the decreased vessel density in the deep retinal vascular plexus with the serum creatinine level, the age of diabetes onset, and the disease duration [15,36]. In the next study from the same center, on the larger group (*n* = 175) age of the onset of T1D correlated significantly positively with foveal thickness (FT), and negatively with FAZ [39]. The mean HbA1c was negatively associated with decreased superficial retinal vascular plexus density, and foveal and parafoveal thicknesses. The authors observed a weak association between FT and children’s weight and height, but not with BMI or BMI Z-score. Interestingly, the study revealed the effect of diabetes ketoacidosis at the onset of T1D, measured by pH, on OCT parameters. Another interesting influence observed in this study was the statistically significant correlations between the duration of continuous subcutaneous insulin infusion (CSII) treatment and negatively FAZ, and positively FT and foveal superficial plexus vessel density. After dividing the group according to the type of treatment (MDI = 81 or CSII = 87), the authors recorded the differences in FAZ, and the parameters of superficial and deep vessel plexuses densities, but not retinal thickness. Such changes may indicate early microcirculatory disorders in children with type 1 diabetes, but further studies are needed to determine if the results may influence the incidence of DR in future. Kara and Can found significantly lower foveal vessel density in the group of diabetic children compared with the controls and concluded that OCTA can be used for the early detection of DR in youths [40]. Similar conclusions were made by Veiby et al., after they confirmed lower superficial and deep capillary vessel densities in 315 T1D eyes [41]. Demir et al. emphasized that in children and adolescents with T1D, without signs of DR, vessel density in the optic disc region is affected earlier than in the macular region [42]. Onoe et al. in youths with T1D showed that a larger FAZ area was associated with higher annual HbA1c, more episodes of severe hypoglycemic attacks and an older onset age [43].

Niestrata-Ortiz et al. have also used the OCT for a large pediatric group with T1D, to reveal enlargement of FAZ, which may indicate early subclinical angiopathy [44]. The authors have also noticed that choroidal thickening correlates with the duration of diabetes, suggesting hypoxic reactive vasodilatation. The results of other studies so far indicated choroidal thinning in patients with DR [37]. The influence of puberty on the risk of DR has been known for many years, and has had an impact on the recommendation of early screening in pubertal and post-pubertal groups of adolescents. The analysis of the OCT and OCTA results confirmed this, showing statistically significant differences in FT and the foveal superficial capillary plexus in prepubertal and pubertal subgroups [45]. Analyzing the subgroups of the prepubertal and post-pubertal children, the authors observed statistically significant differences between FT, whole SCP, and foveal SCP. The comparison of the pubertal and post-pubertal subjects revealed differences in the parafoveal deep capillary plexus. In the groups matched according to the duration of diabetes, this study found differences between prepubertal, pubertal, and post-pubertal children in FT, PFT, and parafoveal SCP and DCP, confirming the effect of puberty (with the effect of diabetes duration eliminated).

Wysocka-Mincewicz et al. detected significant correlations between thyroid-stimulating hormone (TSH), free triiodothyronine (FT3), free thyroxine (FT4) levels, and OCT and OCTA parameters [46]. If the authors selected the group with coexisting autoimmune thyroiditis, this group had statistically worse results in terms of foveal and parafoveal retinal thickness, ganglion cell complex and global loss volume, emphasizing the strong influence of good thyroid hormone control on the condition of the retina.

## 2. Summary

The use of continuous subcutaneous insulin infusion using insulin pumps with a real-time glucose monitoring system, effective education of young patients and their families, and multi-specialist care have made cases of DR and diabetic cataracts in children extremely rare. However, children should have regular eye examinations to control other complications of diabetes that are not so dangerous for the eyes (recurrent inflammation, meibomian cyst, barley), to correct refractive errors and enable these children to develop properly.

While additional studies are needed before strict recommendations may be made, liberalizing the guidelines for pediatric DR screening may be warranted and purposeful.

Longitudinal studies on the retinal changes in these patients are needed to provide the data needed to confirm the frequency of curable diabetic eye disease and the impact of less rigorous screening. OCT and OCTA utility is very promising due to the possibility to detect disturbances in retinal vessel density, foveal thickness and foveal avascular zone. Alterations in retinal vessel density occur early before the onset of clinically detectable other diabetes-related complications. To the best of our knowledge, all the current reports on OCT and OCTA in the population of adolescents with T1D confirm the usefulness of these methods in the non-invasive assessment of their microvasculature and the detection of early disorders, before the clinical diagnosis of DR.

However, longitudinal observation of these young patients is necessary to determine if any of these OCT and OCTA markers are predictive for future DR occurrence and severity.

## Figures and Tables

**Table 1:** Classification of diabetic retinopathy by Early Treatment of Diabetic Retinopathy Study (ETDRS).

Non-Proliferative Diabetic Retinopathy (NPDR)	
Mild diabetic retinopathy (simple)	microaneurysms only
Moderate non-proliferative retinopathy	more than just microaneurysms, but less than severe non-proliferative retinopathy
Severe non-proliferative diabetic retinopathy	any of the following (governed by the 4/2/1 rule), and no signs of proliferative DR —>20 intra-retinal hemorrhages in each 4 quadrants of retinal circumference, —definite venous beading in at least 2 retinal quadrants, prominent intra-retinal microvascular abnormalities (IRMA) in at least 1 quadrant, —no signs of proliferative retinopathy.
**Proliferative diabetic retinopathy (PDR)**—one or more of the following: neovascularization and/or vitreous or preretinal hemorrhages)	

## Data Availability

Not applicable.
